# Influence of brain atrophy using semiquantitative analysis in [^123^I]FP-CIT single-photon emission computed tomography by a Monte Carlo simulation study

**DOI:** 10.1038/s41598-021-04078-x

**Published:** 2022-01-07

**Authors:** Hiroki Nosaka, Masahisa Onoguchi, Hiroyuki Tsushima, Masaya Suda, Satoshi Kurata, Ayano Onoma, Ryosuke Murakawa

**Affiliations:** 1 Clinical Imaging Center for Healthcare, Nippon Medical School, 1-12-15 Sendagi, Bunkyo, Tokyo, 113-0022 Japan; 2 Department of Quantum Medical Technology, Graduate School of Medical Sciences, Kanazawa University, 5-11-80 Kodatsuno, Kanazawa, Ishikawa 920-0942 Japan; 3 Department of Radiological Technology, Faculty of Health Sciences, Kobe Tokiwa University, 2-6-2 Otanicho Nagata-ku, Kobe, Hyogo 653-0838 Japan; 4 Department of Radiological Sciences, Ibaraki Prefectural University of Health Sciences, 4669-2, Ami, Ibaraki 300-0394 Japan; 5 Department of Radiology, Nippon Medical School, 1-1-5 Sendagi, Bunkyo, Tokyo, 113-8602 Japan; 6 Department of Radiological Technology, Ibaraki Prefectural Central Hospital, 6528 Koibuchi, Kasama, Ibaraki 309-1793 Japan; 7 Department of Radiology, Yokohama City University Hospital, 3-9 Fukuura, Kanazawaku, Yokohama, Kanagawa 236-0004 Japan; 8 Graduate of Radiology, Ibaraki Prefectural University of Health Sciences, 4669-2, Ami, Ibaraki 300-0394 Japan

**Keywords:** Medical research, Molecular medicine, Neurology, Physics

## Abstract

The specific binding ratio (SBR) is an objective indicator of N-ω-fluoropropyl-2β-carbomethoxy-3β-(4-[123I] iodophenyl) nortropane ([^123^I]FP-CIT) single-photon emission computed tomography (SPECT) that could be used for the diagnosis of Parkinson’s disease and Lewy body dementia. One of the issues of the SBR analysis is that the setting position of the volume of interest (VOI) may contain cerebral ventricles and cerebral grooves. These areas may become prominent during the brain atrophy analysis; however, this phenomenon has not been evaluated enough. This study thus used Monte Carlo simulations to examine the effect of brain atrophy on the SBR analysis. The brain atrophy model (BAM) used to simulate the three stages of brain atrophy was made using a morphological operation. Brain atrophy levels were defined in the descending order from 1 to 3, with Level 3 indicating to the most severe damage. Projection data were created based on BAM, and the SPECT reconstruction was performed. The ratio of the striatal to background region accumulation was set to a rate of 8:1, 6:1, and 4:1. The striatal and the reference VOI mean value were decreased as brain atrophy progressed. Additionally, the Bolt’s analysis methods revealed that the reference VOI value was more affected by brain atrophy than the striatal VOI value. Finally, the calculated SBR value was overestimated as brain atrophy progressed, and a similar trend was observed when the ratios of the striatal to background region accumulation were changed. This study thus suggests that the SBR can be overestimated in cases of advanced brain atrophy.

## Introduction

It has been known for over three decades that the striatal dopamine transporter (DAT) density is reduced in dopaminergic degenerative disorders such as Parkinson’s disease (PD) and dementia with Lewy bodies (DLB)^1,2^. The single-photon emission computed tomography (SPECT) imaging of DAT density with N-ω-fluoropropyl-2β-carbomethoxy-3β-(4-[123I]iodophenyl)nortropane ([123I]FP-CIT) is a novel imaging modality commonly used in clinical settings nowadays. The [123I]FP-CIT imaging is a recently developed tool that can prove to be extremely useful in the diagnosis of dopaminergic degenerative disorders^3–5^ because it can differentiate these disorders from other neurodegenerative diseases such as Alzheimer’s disease (AD).

A very common method to evaluate the reduction in DAT density with [123I]FP-CIT SPECT, is to perform semi-quantitative analyses using the specific binding ratio (SBR)^6,7^. This ratio could be a prominent index of differential diagnosis, thus helping clinicians selects the appropriate patient treatment. First proposed by Bolt in 2006, it is one of the most useful and established quantitative indices among a plethora of analytical strategies in this field^8^.

More specifically, the SBR is calculated as the ratio of specific binding counts from the striatal volume of interest (VOI) to the nonspecific binding counts from a reference VOI, devoid of DAT. The equation of SBR for this study is

1 $${\text{SBR}} = \left\{ {\frac{{{\text{Cs}}_{{{\text{total}}}} }}{Cr} - {\text{Vs}}_{{{\text{VOI}}}} } \right\}\left( \frac{1}{Vs} \right),$$

where SBR is the specific binding ratio by Bolt, *Cs*_*total*_ is the striatal VOI total counts [counts], *Cr* represents the mean counts per volume in the reference VOI [counts/cm^3^], *Vs*_*VOI*_ is the volume of striatal VOI [cm^3^], and *Vs* is the volume of the striatum [cm^3^]. The use of a large VOI for striatum is recommended to minimize the influence of the partial volume effect^8^.

Patient’s brain atrophy is one of the issues encountered when using SBR in clinical practice. Enlargement of cerebral ventricles and sulci in patients with brain atrophy is a common issue in diagnostic imaging. It is known that many degenerative brain diseases are accompanied by brain atrophy. Atrophy of the hippocampus is a characteristic of dementias, such as AD and DLB. Additionally, atrophy of the medial temporal lobe is known to be present in AD and DLB^9^. In a longitudinal study using magnetic resonance imaging (MRI), DLB, and AD, the authors reported greater rates of whole brain atrophy compared to age-matched normal groups^10^. A large striatal VOI for SBR analysis may contain several low-accumulation areas because of the adjacent cerebral ventricles and sulci. Consequently, contamination of these low-accumulation areas has been reported to decrease the accuracy of SBR^11–15^. Thus, the SBR analysis in case of brain atrophy may lead to decreased diagnostic accuracy.

Several studies have performed SBR calculations using digital phantoms^13–15^. However, these studies performed simple numerical calculations and did not sufficiently consider the clinical situation. Therefore, this study used a digital phantom and a Monte Carlo simulation, which can be employed for more clinically relevant studies because it can consider the effects of physical phenomena such as photon scattering and attenuation^16^.For this reason, this study investigated the effect of brain atrophy on SBR using a Monte Carlo simulation.

## Methods

### Brain atrophy model (BAM)

A BAM was generated from the Zubal brain digital phantom provided by Yale University, USA^17–19^. Since the Zubal head phantom is based on high-resolution MRI images (width: 256 pixels, height: 256 pixels, thickness: 128 pixels, voxel size: 1.1 × 1.1 × 1.4 mm) of normal adults, the derived BAM could reproduce detailed clinical studies.

The BAM was created out of the following four segments extracted from the Zubal phantom. The bone regions were generated from the skull; the striatal regions were putamen and caudate nuclei extracted from the phantom; the cerebral ventricle regions were the third, fourth, and lateral ventricles, but also the cerebral aqueduct, and the spinal cord. Furthermore, and with respect to the brain parenchyma regions, white and gray matter was extracted from the phantom. Regions from the cerebral ventricles were also extracted to generate homogeneous brain parenchyma.

Background regions were created by subtracting cerebral ventricle regions from brain parenchyma . These background regions were used as reference regions for [123I]FP-CIT SPECT nonspecific binding counts. Additionally, cerebral ventricle and brain parenchyma regions underwent a morphological operation to reproduce brain atrophy using the image-processing software ImageJ as described below^20,21^.

**Figure 1:**
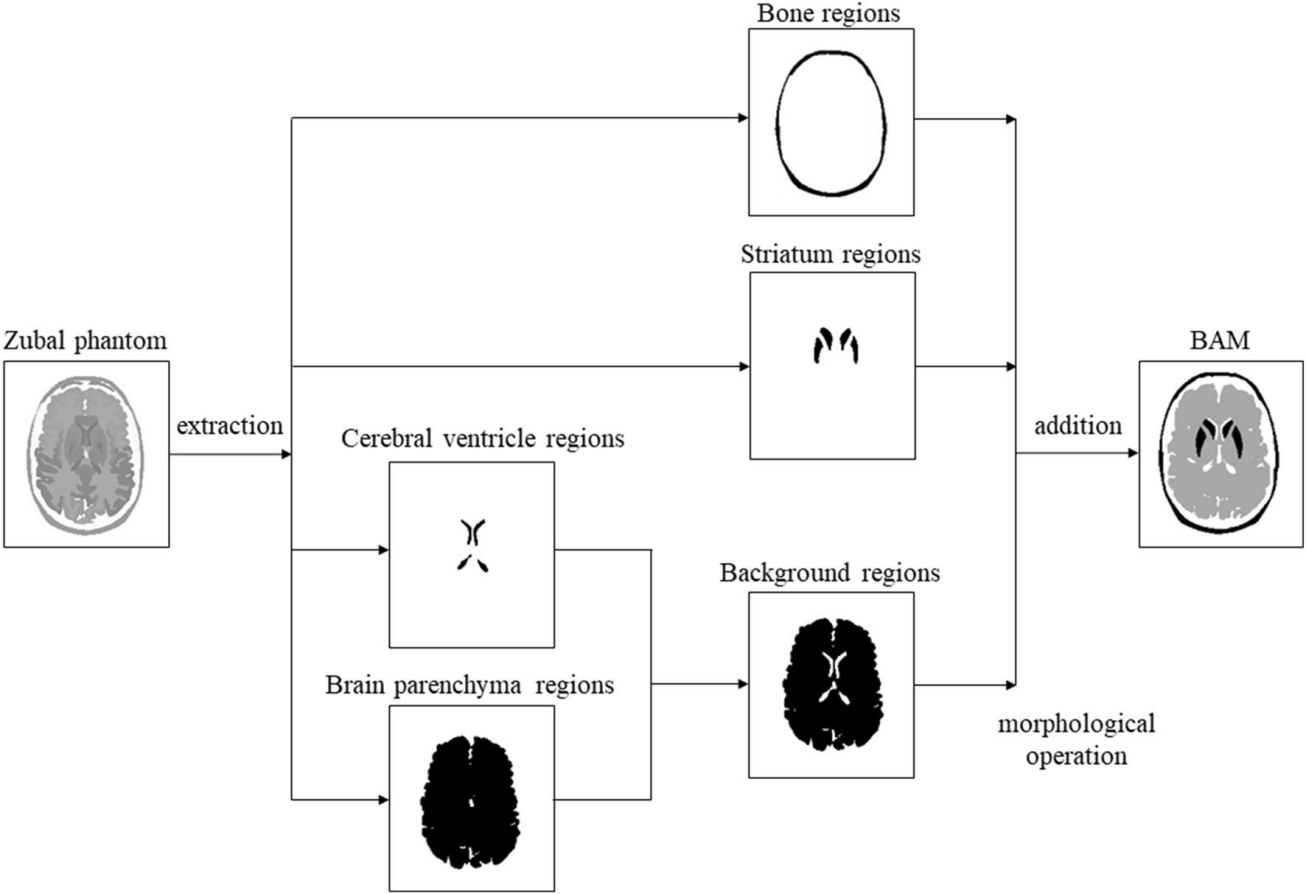
At first, four areas were extracted from the magnetic resonance imaging based Zubal head phantom. Next, the background regions were created by differentiating the cerebral ventricle regions from the brain parenchyma regions. Finally, brain atrophy model (BAM) was generated by combining bone, striatum, and background regions subjected to the morphological operation.

Finally, BAM was created by adding the bone, striatum, and background regions. This process is shown in Fig. 1. The same operation was performed on all slices, and thus the process was performed in three dimensions.

**Figure 2:**
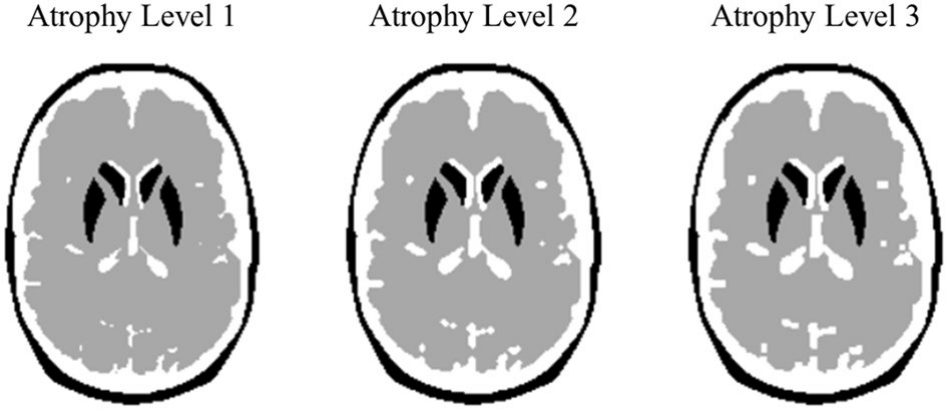
Three BAM patterns were created. Atrophy Level 3 corresponds to the most severe brain damage. By contrast, atrophy Level 1 corresponds to the original Zubal head phantom where no morphological operation is applied.

To simulate the three patterns of brain atrophy, the background regions underwent a morphological operation with 0.5 pixels. The ratio of the striatum to brain parenchyma accumulation was set to a rate of 8:1, 6:1, and 4:1. Brain atrophy levels were defined in the descending order from 1 to 3, with Level 3 corresponding to the most severe damage. The generated BAM is shown in Fig. 2. The volume of the whole brain volume at atrophy Level 1, the least atrophic level, was 1387.41 cm^3^. Several studies on brain volume and PD have shown that the whole brain volume of normal control patients [^123^I]FP-CIT SPECT was reported to be approximately < 1500 ml^22,23^. Therefore, Level 1 defined normal cases in this study.

### Monte Carlo simulation

**Figure 3:**
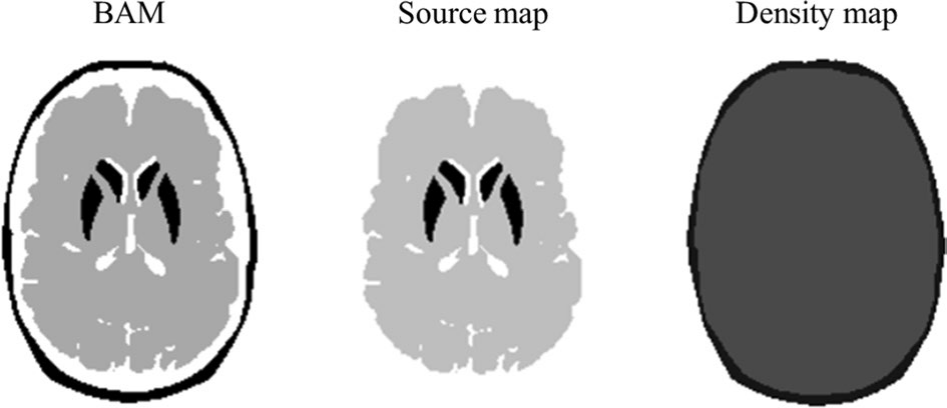
The source and density maps were created from BAM. The source map involved both background and striatal regions. The ratio of the striatal to background region accumulation was set to rates of 8:1, 6:1, and 4:1. The density map consisted of two parts only—bone and non-bone—and each density was 1.22 g/cm3 and 1.04 g/cm3, respectively.

Projection data were generated using the Monte Carlo simulation code SIMIND provided by LUND University, Sweden^16,24–31^. This code considers photoelectric, incoherent, coherent interactions, and pair production in the detector and phantom. The source and the density maps were developed from the BAM to simulate the projection date. Assuming a simple model, the source map was only in the background and striatal regions and no sources were placed in any other regions. The background regions were set to 3.60 kBq/ml, which is the amount of accumulation observed after 5 h of injection in clinical settings^1^. The ratio of the striatal to background region accumulation was set to a rate of 8:1, 6:1, and 4:1. The density map was created with bone and non-bone regions, and the density of each region was 1.22 g/cm^3^ and 1.04 g/cm^3^ simulated clinical values, respectively. The source map and the density map are shown in Fig. 3.

Simulations were performed while assuming a Gamma-Camera detector structure such as the one designed for the BrightView XCT(Hitachi, Ltd. Tokyo, Japan). The BrightView XCT is provided with a dual NaI crystal detector that is optimized for size (field of view: 40.6 × 53.9 cm: thickness: 9.5 mm). A low-energy high-resolution collimator was also simulated, and a 7.0 cm photomultiplier tube was coupled with the NaI detector. Finally, ^123^I emissions were simulated as a radiation source.

Projection data were simulated as a matrix of 128 × 128 pixels, and they were collected using 90 projections with a 4° step size. The sum of all projection data was adjusted to 1.5 million counts, and the pixel size was set to 3.2 mm. The main energy window was maintained within the 143.1 to 174.9 keV range, and its value was 159 keV ± 10%. Five sets of data were simulated per condition. The conditions for these simulated projection data were determined by referring to the Japanese [123I] FP-CIT clinical diagnosis guidelines^32^. A maximum of five scatter orders were permitted in the phantom. For all simulations, projection data were simulated such that they corresponded to an acquisition time of 30 s. Five independent simulations were performed for each condition.

### Image reconstruction

The simulated projection data were reconstructed using the Prominence Processor software, version 3.1. Reconstruction conditions were determined in accordance with the Japanese [123I]FP-CIT clinical diagnostic guidelines^32^. Filtered back-projection was performed with a Butterworth filter, using a cutoff frequency of 0.5 cycles/cm and an order of eight. Attenuation and scatter corrections were not performed.

### Image analysis

**Figure 4:**
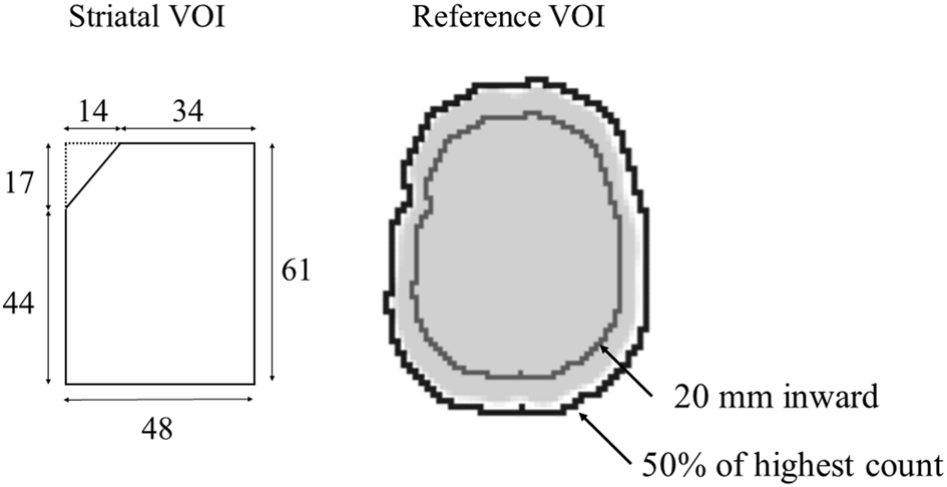
Schematic diagram of VOI setting. The unit of the striatal VOI is mm. The reference VOI was set to background regions, and it was then smoothed except for the striatal VOI.

The reconstructed images were analyzed using the image analysis software DatView (Nihon Medi-Physics, Japan). This software allows the SBR computations based on the Bolt analysis method^8^. Quantification was performed by automatically aligning all images to the anterior commissure–posterior commissure line. Since the BAM set was based on the same Zubal phantom, the inclination of the brain did not vary within the set. Thus, brain inclination corrections were applied across the used models. The striatal VOI volume (Vs_VOI_) and striatal volume (Vs) were 141.56 and 17.92 cm^3^ respectively, which were intrinsic values in the analysis software, and the same values were used for all SBR measurements. The threshold value for the reference VOI was determined based on the 50% of the highest count value excluding the striatal area. To exclude the margins with partial volume effects, the borders of the reference area were set to 20 mm inward from the VOI margins. All analysis parameters were selected following the Bolt protocol. The schematic diagram of the VOI setting is shown in Fig. 4.

The SBR quantification was performed on all simulated images for each of the right and left striatal VOI, and five independent counts were averaged per condition. The average value across the right and left sides of the brain was taken as the output measure.

Significance difference tests were performed using Student’s t-test. Statistical significance was defined as a *p*-value < 0.05. A significance difference test for each level was performed using Level 1 as the standard.

## Results

### BAM

Table 1 shows the volume of BAM background regions created using the subsequent morphological operation. As the brain atrophy progresses, the volume of the background regions decreased. Atrophy Level 1 corresponds to the original Zubal head phantom with no morphological operation applied and to the normal total brain volume in clinical practice^22,23^.

**Table 1:** Changes in the brain atrophy level and its volume.

Atrophy level	Morphological operation	BG region [cm^3^]
1	No processing	1387.41
2	− 0.5 pixel	1285.63
3	− 1.0 pixel	1243.29

Background regions changed based on Level 1. The higher the level, the greater the brain atrophies.

### Monte Carlo simulation

**Figure 5:**
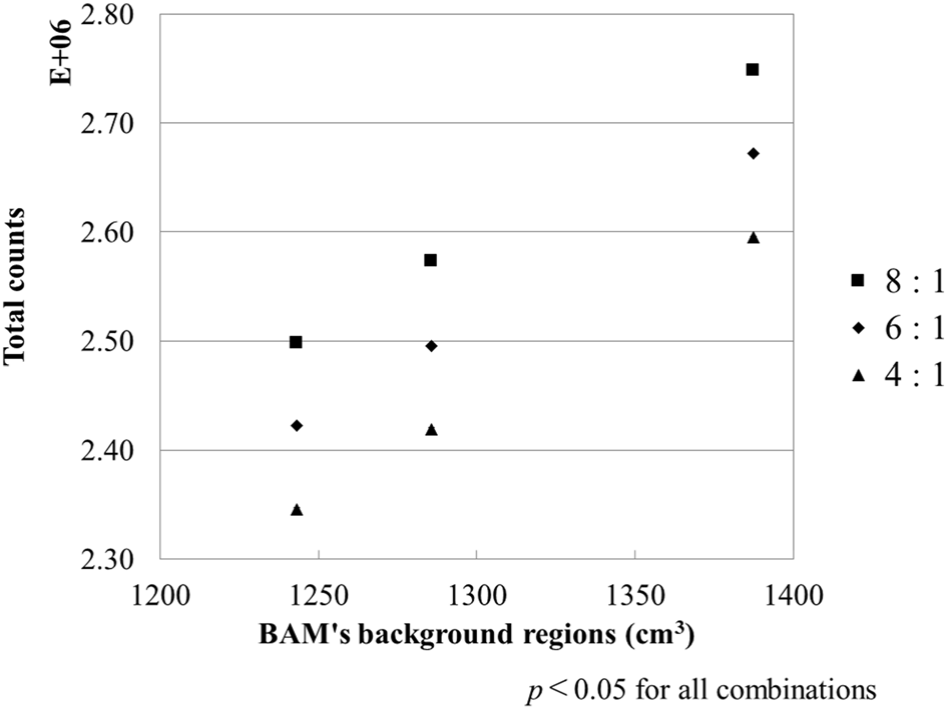
Relationship between detected total counts and the volume of BAM background regions. Cs decreased linearly with the increasing volume of BAM background regions.

The total photon number per projection of the source map was between 770,118 and 902,673 counts. Figure 5 shows a total number of photons detected by the detector. All combinations of total detected photons were significantly different (*p* < 0.05).

### Striatal and reference VOI value

**Figure 6:**
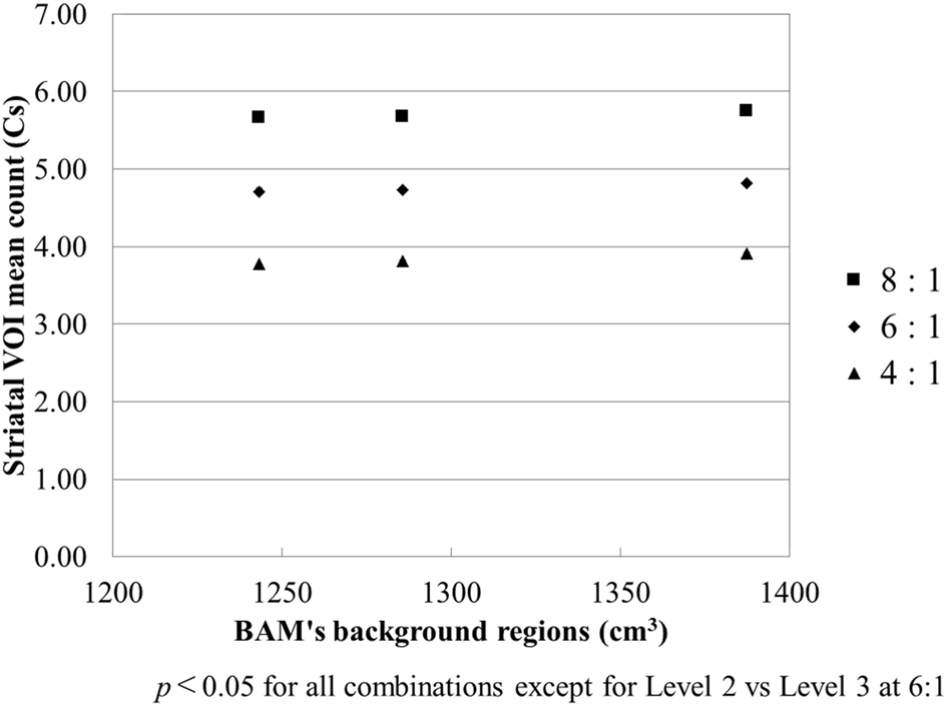
Relationship between Cs and the volume of BAM background regions. Cs decreased linearly with the increasing volume of BAM background regions.

**Figure 7:**
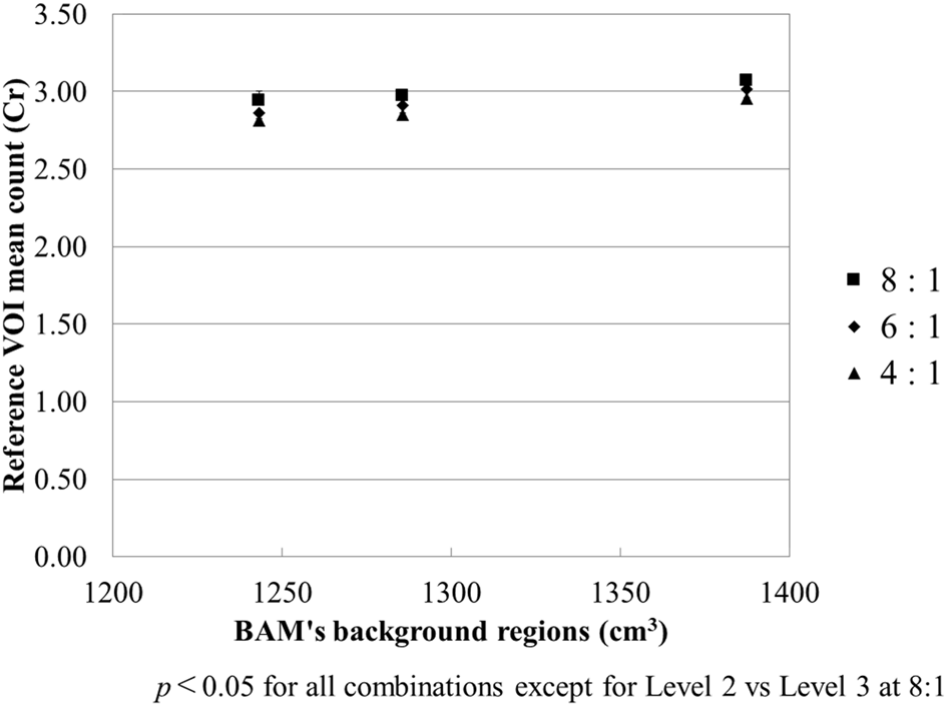
Relationship between Cr and the volume of BAM background regions. Cr decreased linearly with the increasing volume of BAM background regions.

Figure 6 illustrates the relationship between Cs and the volume of the BAM background regions, whereas Fig. 7 presents the relationship between Cr and the volume of the BAM background regions. The minimum and maximum errors are also listed. These figures demonstrate that both Cs and Cr have a strong positive correlation with the volume of the BAM background regions. There was no difference in the trend between the differences in the ratio of the striatal to background region accumulation. The slopes of the approximate curves for Cs with the BAM background regions were 0.0007 (R^2^ = 0.9842) for 8:1, 0.0007 (R^2^ = 0.9956) for 6:1, and 0.001 (R^2^ = 0.9984) for 4:1. Similarly, the slopes for Cr were 0.0009 (R^2^ = 0.9976) for 8:1, 0.0011 (R^2^ = 0.9986) for 6:1, and 0.001 (R^2^ = 0.9998) for 4:1. All combinations of Cs except for Level 2 vs Level 3 at 6:1 differed significantly (*p* < 0.05). And all combinations of Cr except for Level 2 vs Level 3 at 8:1 differed significantly (*p* < 0.05).

### SBR

**Figure 8:**
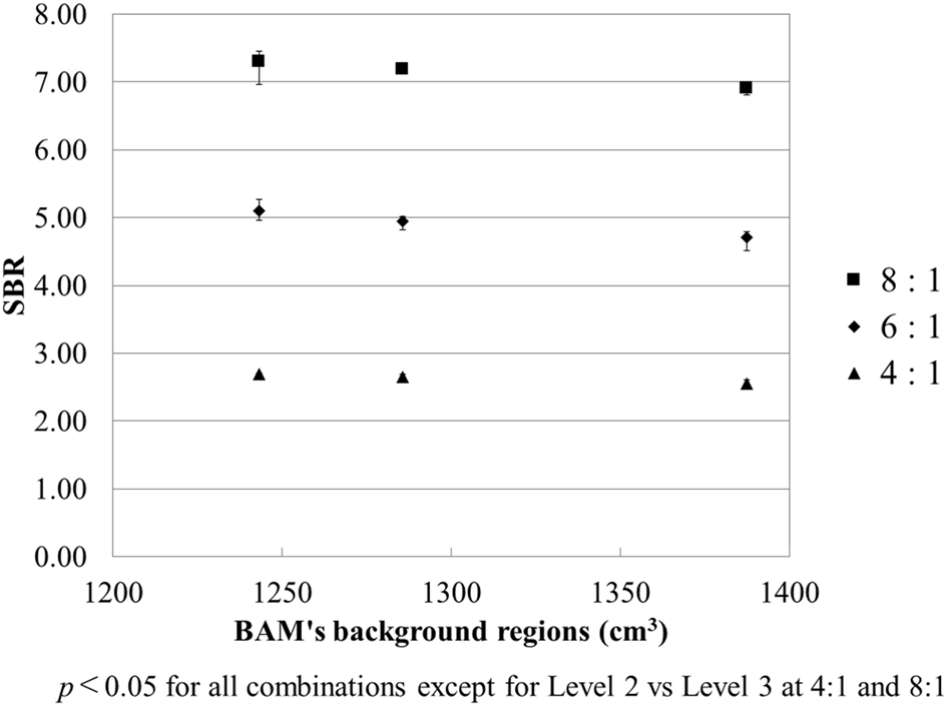
Relationship between specific binding ratio (SBR) and the volume of BAM background regions. The SBR decreased with the increasing volume of BAM background regions.

Figure 8 exhibits the relationship between the SBR and the volume of BAM background regions. These values were inversely correlated, that is, the SBR tended to be overestimated with increasing brain atrophy and decreasing BAM background regions. The trend did not change for different ratios of the striatal to background region accumulation. The slopes of the approximate curves for SBR and the BAM background regions were − 0.0028 (R^2^ = 0.9998) for 8:1, − 0.0027 (R^2^ = 0.9829) for 6:1, and − 0.0009 (R^2^ = 0.9551) for 4:1. All combinations of SBR except for Level 2 vs Level 3 at 4:1 and 8:1 differed significantly (*p* < 0.05).

## Discussion

This study used Monte Carlo simulations to investigate the effect of brain atrophy on SBR. Different degrees of brain atrophy severity were modeled by the morphological operation to vary the volumes of the brain parenchyma and cerebral ventricles. Using this method, it was possible to determine the volume of the brain in detail. Consequently, knowing the volume of the brain allowed us to successfully investigate the relationship between brain atrophy and SBR.

Cs was significantly correlated with the volume of the BAM background regions (Fig. 6). More specifically, this measured value decreased as the volume of the brain parenchyma regions decreased. Similarly, Cr showed a significant correlation with the brain parenchyma regions (Fig. 7). As the volume of the brain parenchyma regions decreased, the counts of the reference VOI decreased. These phenomena are usually attributed to the relative increase in the cerebral sulci and ventricle region volumes in each VOI caused by brain atrophy. To minimize the influence of the partial volume effect, the large striatal VOI is used for calculating the SBR in the Bolt method. Thus, in brain atrophy cases, it is possible that low-count regions where [123I]FP-CIT does not accumulate, such as the cerebral sulci and ventricles, may be included within the striatal VOI. It is considered that this contamination by low-count regions can result in decreased counts in the striatal VOI. Similarly, it is considered that the reference VOI value also decreased due to the contamination by low-count regions caused by brain atrophy.

Change in the SBR value can be explained through the SBR calculation formula. In the SBR computation, the striatal VOI (Vs_VOI_) volume and the striatum (Vs) volume were kept constant across the simulations (Vs_VOI_ and Vs were 141.56 and 17.92 cm^3^, respectively). Therefore, Vs_VOI_ and Vs did not affect the SBR analysis. Consequently, only the striatal VOI total counts (Cs_total_) and the mean counts per volume in the reference VOI (Cr) affected the output value in Eq. (1). Hence, the ratio Cs_total_/Cr becomes the critical parameter for the SBR calculation. Since the striatal VOI (Vs_VOI_) volume is always constant, both Cs_total_ and Cs follow the same trend. Therefore, the Cs value was used in this study. Even when the actual Cs and Cr values change, the measurements will remain constant provided that the rate of change is the same. However, in this study, the SBR was overestimated due to brain atrophy because the rate of change of Cs and Cr was different. Table 2 shows the rate of change of Cs and Cr and the resulting SBR. This table shows the rate of increase in the values at each level, with Level 1 as the reference.

**Table 2:** Rate of change in values due to brain atrophy.

Atrophy Level	Striatal VOI mean counts (Cs)			Reference VOI mean counts (Cr)			SBR		
	8:1	6:1	4:1	8:1	6:1	4:1	8:1	6:1	4:1
1	100.00	100.00	100.00	100.00	100.00	100.00	100.00	100.00	100.00
2	98.67	98.31	97.34	96.85	96.52	96.52	104.02	104.95	103.47
3	98.38	97.80	96.42	95.81	94.82	95.16	105.80	108.40	105.40

Rate of change for each value with reference to atrophy Level 1. All values increased with the increase in the degree of brain atrophy.

We found that the change in Cr is larger than that of Cs. This can be explained by the Bolt analysis procedure^8^. In the Bolt analysis, when the reference VOI is initially set, smoothing is performed within the reference regions, thus excluding the striatal VOI. This smoothing process might be affecting the decreasing rate of change for Cr. As a result, the SBR was overestimated because changes in Cs were smaller than changes in Cr. Furthermore, the overestimation of the SBR was similar when the ratios of the striatal to background region accumulation were changed.

This study underlines that the SBR calculated using the Bolt approach showed a significantly negative correlation with brain atrophy. Consequently, the decrease in brain parenchyma volume caused by brain atrophy induces an overestimation of SBR. Our findings are consistent with the results by Furuta et al.^14^ where in the size of the brain ventricles were solely altered.

Furthermore, we found that brain atrophy could particularly affect the reference VOI. Several methods for calculating the SBR rely on a reference VOI, which in turn corresponds to the whole brain or the occipital lobe^6,33^. In the SBR analysis of patients with brain atrophy, the modification of the reference VOI position according to the degree of regional brain atrophy should be considered as a preferable option. However, Watanabe’s report highlighted that the SBR values could change according to the setting of the reference VOI threshold^15^. Thus, it may be precarious to shift the setting range of the threshold value and the VOI location unnecessarily.

Most patients that undergo the [123I]FP-CIT SPECT examination are elderly. It has been reported that old age is a prominent factor of brain atrophy^34,35^. In this study, we showed that brain atrophy induced an overestimation of the SBR values. This overestimation is a confounding factor for the estimation of the DAT density decrease in the diagnosis of dopaminergic degenerative disorders.

The SBR overestimation may be a particular issue in diseases in which the whole striatum accumulation decreases such as DLB. It has been shown that SBR estimates a decrease in DLB with the time course of the [123I]FP-CIT SPECT investigation. Recent data analysis research on ENC-DAT has shown that SBR decreases with age^36,37^. However, in cases with advanced brain atrophy due to aging, this effect may be counteracted by the related SBR overestimation.

Finally, our findings revealed that the accumulation of the striatum was constant. Thus, alternative unbiased models must be considered to properly measure the decreased striatal [123I]FP-CIT SPECT accumulation in diseases such as PD and DLB. Furthermore, one of the limitations of this study is that the whole brain volume was geometrically changed in the morphological operation; thus, fine tuning was not possible. It was also suggested that the detected count may decrease due to brain atrophy. Therefore it might be recommended that imaging conditions be based on acquisition counts rather than acquisition time. This Monte Carlo simulation study clarified that SBR values were overestimated in patients with brain atrophy. Thus, the influence of brain atrophy should be seriously considered when measuring SBR.

## Conclusion

In conclusion, the SBR is overestimated in cases of progressive brain atrophy. It was also found that the SBR overestimates were more affected when striatal accumulation was low.

## Abbreviations

SBR: Specific binding ratio
SPECT: Single-photon emission computed tomography
VOI: Volume of interest
BAM: Brain atrophy model
DAT: Dopamine transporter
PD: Parkinson’s disease
DLB: Dementia with Lewy bodies
Vs_VOI_: Striatal VOI volume
Vs: Striatal volume
Cs: Mean counts per volume for striatal VOI
Cr: Mean counts per volume for reference VOI
Cs_total_: Striatal VOI total counts

## References

[1] Takano K (1999). Phase 1 clinical study of ^123^I-FP-CIT, a new radioligand for evaluating dopamine transporter by SPECT (II): tracer kinetics in the brain. JSNM.

[2] Piggott MA (1998). Nigrostriatal dopaminergic activities in dementia with Lewy bodies in relation to neuroleptic sensitivity: comparisons with Parkinson’s disease. Biol. Psychiatry..

[3] McKeith IG (2005). Diagnosis and management of dementia with Lewy bodies: third report of DLB Consortium. Neurology.

[4] Niznik HB, Fogel EF, Fassos FF, Seeman P (1991). The dopamine transporter is absent in parkinsonian putamen and reduced in the caudate nucleus. J. Neurochem..

[5] McKeith I (2007). Sensitivity and specificity of dopamine transporter imaging with ^123^I-FP-CIT SPECT in dementia with Lewy bodies: a phase III, multicentre study. Lancet Neurol..

[6] Booij J (1998). Imaging of dopamine transporters with iodine-123FP-CIT SPECT in healthy controls and patients with Parkinson's disease. J. Nucl. Med..

[7] Badiavas K, Molyvda E, Iakovou I, Tsolaki M, Psarrakos K, Karatzas N (2011). SPECT imaging evaluation in movement disorders: far beyond visual assessment. Eur. J. Nucl. Med. Mol. Imaging..

[8] Tossici-Bolt L, Hoffmann SM, Kemp PM, Mehta RL, Fleming JS (2006). Quantification of [^123^I]FP-CIT SPECT brain images: an accurate technique for measurement of the specific binding ratio. Eur. J. Nucl. Med. Mol. Imaging..

[9] Tam CW, Burton EJ, McKeith IG, Burn DJ, O'Brien JT (2005). Temporal lobe atrophy on MRI in Parkinson disease with dementia: a comparison with Alzheimer disease and dementia with Lewy bodies. Neurology.

[10] Middelkoop HA (2001). Dementia with Lewy bodies and AD are not associated with occipital lobe atrophy on MRI. Neurology.

[11] Mizumura S (2018). Improvement in the measurement error of the specific binding ratio in dopamine transporter SPECT imaging due to exclusion of the cerebrospinal fluid fraction using the threshold of voxel RI count. Ann. Nucl. Med..

[12] Hayashi T, Mishina M, Sakamaki M, Sakamoto Y, Suda S, Kimura K (2019). Effect of brain atrophy in quantitative analysis of 123I-ioflupane SPECT. Ann. Nucl. Med..

[13] Washimi M, Yamaki N, Yanagisawa M (2016). A study on the effect by ventricles on quantitative index in ^123^I-Ioflupane SPECT images. JSNMT.

[14] Furuta A, Onishi H, Nakamoto K (2017). Development of realistic striatal digital brain (SDB) phantom for ^123^I-FP-CIT SPECT and effect on ventricle in the brain for semi-quantitative index of specific binding ratio. JSRT.

[15] Watanabe A, Inoue Y, Asano Y, Kikuchi K, Miyatake H, Tokushige T (2017). Examination of a method to determine the reference region for calculating the specific binding ratio in dopamine transporter imaging. JSRT.

[16] Buvat I, Cactiglioni I (2002). Monte Carlo simulations in SPET and PET. Q J. Nucl. Med..

[17] Zubal IG, Harrell CR (1992). Voxel based monte carlo calculations of nuclear medicine images and applied variance reduction techniques. Image Vis. Comput..

[18] Zubal IG, Harrell CR, Esser PD (1990). Monte Carlo determination of emerging energy spectra for diagnostically realistic radiopharmaceutical distributions. Nucl. Instrum. Methods Phys. Res., Sect. A.

[19] Zubal IG, Harrell CR, Smith EO, Rattner Z, Gindi G, Hoffer PB (1994). Computerized three-dimensional segmented human anatomy. Med. Phys..

[20] Rasband, W.S. ImageJ, U. S. National Institutes of Health, Bethesda, Maryland, USA, http://imagej.nih.gov/ij/ (1997–2012).

[21] Schneider CA, Rasband WS, Eliceiri KW (2012). NIH Image to ImageJ: 25 years of image analysis. Nat. Methods.

[22] Xu X (2018). Brain atrophy and reorganization of structural network in Parkinson's disease with hemiparkinsonism. Front. Hum. Neurosci..

[23] Guevara C, Bulatova K, Barker GJ, Gonzalez G, Crossley NA, Kempton MJ (2016). Whole-brain atrophy differences between progressive supranuclear palsy and idiopathic Parkinson’s disease. Front Aging Neurosci..

[24] Ljungberg M, Strand SE (1989). A Monte Carlo program for the simulation of scintillation camera characteristics. Comput. Methods Progr. Biomed..

[25] Toossi MTB, Islamian JP, Momennezhad M, Ljungberg M, Naseri SH (2010). SIMIND Monte Carlo simulation of a single photon emission CT. J. Med. Phys..

[26] The Japanese Society of Radiolodical Technology’s sub group Nuclear Medicine Section. ABC book of experiment in nuclear medicine for beginners. 94–111 (JSRT, 2016).

[27] Shirakawa S (2013). Construction of the quantitative analysis environment using Monte Carlo simulation. JSNMT.

[28] Koch W, la Bartenstein P, Fougère C (2014). Radius dependence of FP-CIT quantification: a Monte Carlo-based simulation study. Ann Nucl Med..

[29] Okuda K (2021). Validation of simulation codes for nuclear imaging using digital phantoms. JSRT.

[30] Okuda, K. *et al*. Calibrated scintigraphic imaging procedures improve quantitative assessment of the cardiac sympathetic nerve activity. Sci. Rep. 10 (2020). 10.1038/s41598-020-78917-8 PMC7736873 33318541

[31] Ito T (2021). Verification of phantom accuracy using a Monte Carlo simulation: bone scintigraphy chest phantom. Radiol. Phys. Technol..

[32] Japan Society of Nuclear Medicine, The Japanese Council of Nuclear Neuroimaging, editors. Ioflupane Clinical Practice Guidelines Second Edition [Translated from Japanese.]. 3–18 (2017).

[33] Tatsch K (1997). Relationship between clinical features of Parkinson’s disease and presynaptic dopamine transporter binding assessed with [123I] IPT and single-photon emission tomography. Eur. J. Nucl. Med..

[34] Ito M, Hatazawa J, Yamaura H, Matsuzawa T (1981). Age-related brain atrophy and mental deterioration-A study with computed tomography. Br. J. Radiol..

[35] Takeda S, Matsuzawa T (1984). Brain atrophy and mental deterioration. Sci. Rep. Res. Inst. Tohoku Univ-C.

[36] Varrone A (2013). European multicentre database of healthy controls for [123I]FP-CIT SPECT (ENC-DAT): age-related effects, gender differences and evaluation of different methods of analysis. Eur. J. Nucl. Med. Mol. Imaging..

[37] Nobili F (2013). Automatic semi-quantification of [123I]FP-CIT SPECT scans in healthy volunteers using BasGan version 2: results from the ENC-DAT database. Eur. J. Nucl. Med. Mol. Imaging..

